# Association of Native American, European, and African Ancestry with CYP450 Pharmacogenetics and Metabolic Phenotypes in Admixed Latin American Populations from Ecuador and Nicaragua

**DOI:** 10.3390/ph19091432

**Published:** 2026-09-10

**Authors:** Carla González de la Cruz, Catalina Altamirano-Tinoco, Caíque Manóchio, Juan Antonio Villatoro-García, Fernando de Andrés, Carmen Mata-Martín, Fernanda Rodrigues-Soares, Pedro Dorado, Eva M. Peñas-LLedó, Ronald Ramírez-Roa, Enrique Teran, Adrián LLerena, RIBEF-IBEROFEN Consortium

**Affiliations:** 1 RIBEF/SIFF Red y Sociedad Iberoamericana de Farmacogenética y Farmacogenómica, 06080 Badajoz, Spain; carla.gonzalezd@unex.es (C.G.d.l.C.); catalina.altamirano76@gmail.com (C.A.-T.); caique.manochio@hotmail.com (C.M.); javillatoro@unex.es (J.A.V.-G.); fernando.deandres@uclm.es (F.d.A.); mariadelcarmen.mata@salud-juntaex.es (C.M.-M.); fernanda.soares@uftm.edu.br (F.R.-S.); pdorado@unex.es (P.D.); elledo@unex.es (E.M.P.-L.); ramirezjuanronald@gmail.com (R.R.-R.); 2 University Institute for Bio-Sanitary Research of Extremadura (INUBE), 06080 Badajoz, Spain; 3 Faculty of Medicine and Health Sciences, University of Extremadura, 06080 Badajoz, Spain; 4 Facultad de Ciencias Médicas, Universidad Nacional Autónoma de Nicaragua—León (UNAN-León), 68, León 21000, Nicaragua; 5 Department of Pathology, Genetics and Evolution, Universidade Federal do Triângulo Mineiro, Uberaba 38025-440, Brazil; 6 Department of Genetics, Ecology and Evolution, Universidade Federal de Minas Gerais, Belo Horizonte 31270-901, Brazil; 7 Unit of Pharmacogenetics and Personalized Medicine, Department of Clinical Pharmacology, Badajoz University Hospital (HUB), Extremadura Health Service (SES), 06080 Badajoz, Spain; 8 Facultad de Odontología, Universidad Americana, Managua 14166, Nicaragua; 9 Colegio de Ciencias de la Salud, Universidad San Francisco de Quito, Quito 170901, Ecuador

**Keywords:** pharmacogenetics, genomic ancestry, CYP450, admixture, genotype-phenotype discordance, metabolic ratio, precision medicine, Ecuador, Nicaragua, Native American ancestry

## Abstract

**Background/Objectives**: Interethnic variability significantly influences drug response, highlighting the importance of assessing genomic ancestry for personalized medicine. Although Latin American populations are highly admixed, they remain underrepresented in pharmacogenetic research. This study examined the association between genomic ancestry, *CYP450* genetic variation, and metabolic phenotypes in Ecuadorian (ECU) and Nicaraguan (NIC) populations. **Methods**: A total of 236 healthy volunteers were studied. Genomic ancestry was evaluated using 83 ancestry-informative markers comprising European (EUR), Native American (NAT), and African (AFR) ancestral components. Genetic variants in *CYP2D6*, *CYP2C9*, *CYP2C19*, *CYP3A4*, and *CYP1A2* were genotyped using TaqMan^®^ assays. Enzyme metabolic capacity was assessed using the CEIBA cocktail-based on five test drugs. and expressed as the logarithm of the metabolic ratio (logMR). **Results:** The combined genomic ancestry composition was 48.16% NAT, 40.40% EUR and 11.40% AFR. EUR ancestry was associated with CYP2C9*2 (adjusted *p-* =0.049), CYP2C19*2 (adjusted *p* = 0.029), and CYP2D6*41 (adjusted *p* = 0.010) alleles. AFR ancestry was associated with CYP2D6*17 (adjusted *p* = 0.001) and CYP2D6*4 (adjusted *p* = 0.132) alleles. Regarding genotype-predicted metabolic phenotypes, EUR ancestry was associated with CYP2C9 gIM (adjusted *p* = 0.033), whereas AFR ancestry with CYP2D6 gIM (adjusted *p* = 0.047) and gPM (adjusted *p* = 0.047). Furthermore, ancestry-related differences in measured metabolic capacity were observed: AFR ancestry was associated with higher logMR values for, CYP1A2, and CYP3A4, while EUR ancestry was associated with higher logMR values for CYP2C9. Overall, substantial overlap in measured metabolic activity (logMR) was observed across genotype-predicted groups for all genes, although some differences were identified. **Conclusions:** Genomic ancestry was associated with the distribution of CYP450 alleles and genotype-predicted metabolizer categories in Ecuadorian and Nicaraguan populations. However, both ancestry and genotype-based classifications showed limited ability to explain interindividual variability in measured metabolic activity, highlighting the complexity of CYP450 phenotype prediction.

## 1. Introduction

Interethnic variation in drug response is well documented. These differences arise from the combined influence of socioeconomic disparities and genetic diversity among populations, including pharmacogenomic variants such as single nucleotide polymorphism and copy-number variations. The problem is further exacerbated by the underrepresentation of diverse populations in genomic research and clinical trials, which limits the generalizability of research findings and hinders the equitable implementation of precision medicine [1]. As one of the most genetically heterogeneous regions in the world, Latin America has been shaped by extensive admixture among Native American (NAT), European (EUR) (mostly Iberians), and African (AFR) populations [2,3]. Nevertheless, genomic data and pharmacogenomic evidence from these populations remain limited, restricting the broader application of precision medicine in the region [3].

This limitation has been highlighted as a major challenge for global pharmacogenomics, particularly in admixed populations where ancestry-related and population-specific variants contribute to pharmacogenetic diversity [4].

The Ibero-American Network of Pharmacogenetics and Pharmacogenomics (RIBEF) has demonstrated that genetic variants involved in pharmacokinetic and pharmacodynamic pathways, particularly those affecting cytochrome P450 (CYP450) enzymes, exhibit population-specific distributions that are strongly associated with genomic ancestry in Latino-American populations [5,6]. These distributions reflect the complex demographic history of the region, characterized by varying contributions of Native American, European, and African ancestries resulting from historical migration and admixture processes [1,2,3,7]. Consequently, genomic ancestry may contribute to interindividual variability in drug response and susceptibility to adverse drug reactions [5,6]. Therefore, understanding the population substructure and the relative contribution of ancestral components is essential for characterizing the genetic diversity of Ecuadorian and Nicaraguan populations and for supporting the development of ancestry-informed pharmacogenetic implementation strategies.

The Ecuadorian population is inherently multicultural and multiethnic, shaped by successive migratory events and sustained admixture. As a result, it reflects successive admixture events involving EUR, NAT and AFR populations, giving rise to substantial ethnic diversity [5,6]. Currently, Ecuador (ECU) comprises three major ethnic groups: mestizo, NAT, and Afro-Ecuadorian. The mestizo group of mixed EUR and NAT ancestry accounts for approximately 85% of the national population [8].

The NIC population is composed largely of mestizos, the result of admixture among EUR, NAT, AFR [6], and, to a lesser extent, Asians, as well as descendants of EUR, zambos, mulattoes, Afro-descendants, and NAT groups [9]. This process of mestizaje emerged during the 16th-century colonization, when the NAT population experienced a marked demographic decline, and the survivors intermingled with Spanish settlers and enslaved sub-Saharan Africans [10].

This study aimed to characterize genomic ancestry in admixed populations from ECU and NIC and to evaluate its association with *CYP450* genetic variation and genotype-predicted metabolic phenotypes, and measured enzyme activity. By focusing on populations that remain underrepresented in pharmacogenetic research, this work seeks to expand current knowledge on ancestry-related variability in drug metabolism and to support the development of ancestry-informed pharmacogenetic implementation strategies for precision medicine in Latin America.

## 2. Results

### 2.1. Genomic Ancestry Characterization

**Figure 1:**
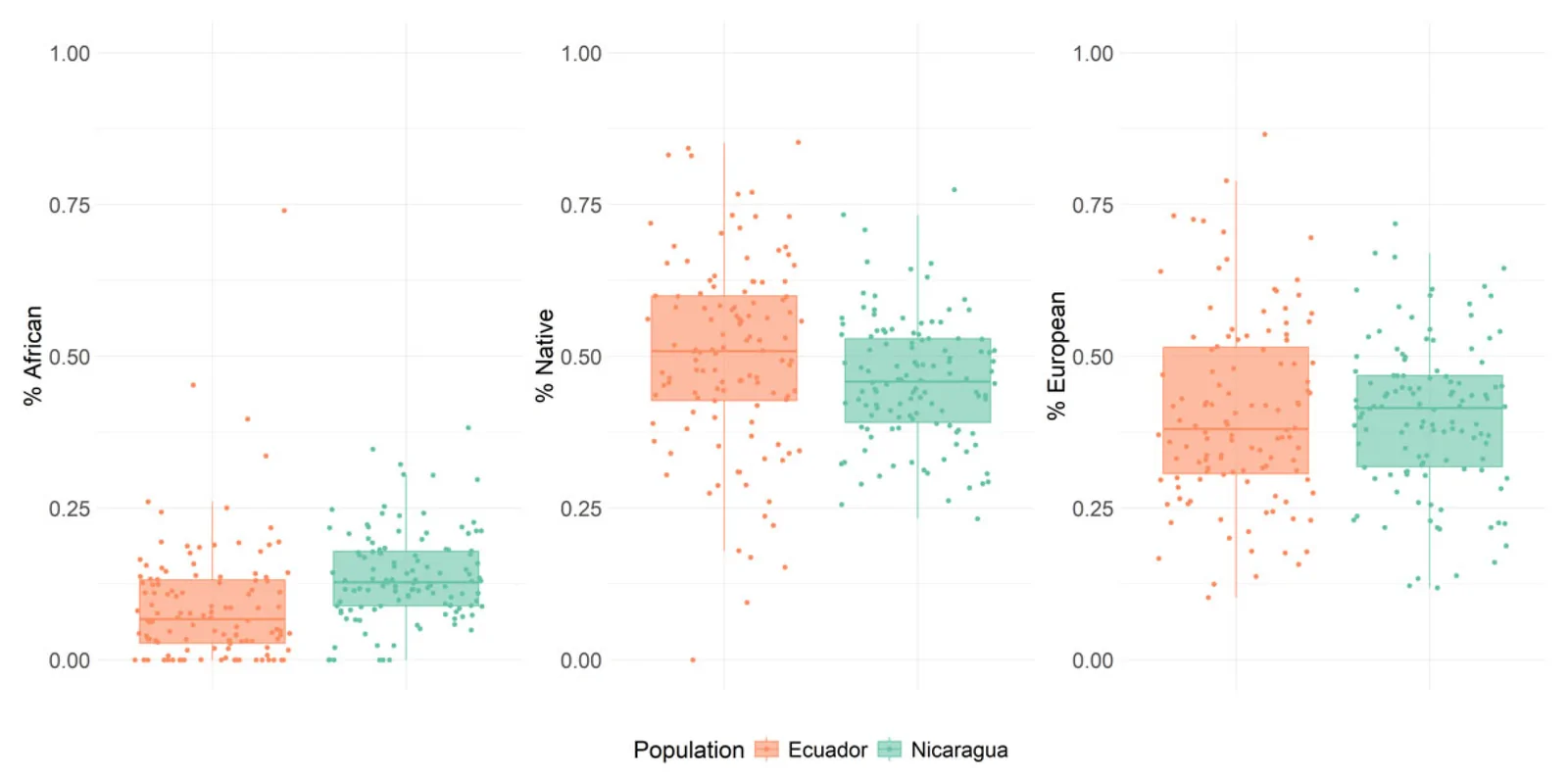
Proportion of genomic African, Native and European ancestral components in population studied from Ecuador and Nicaragua (n = 236 healthy volunteers).

The ECU population exhibited a genomic ancestry composition of 40.62% EUR, 9.10% AFR, and 50.28% NAT. Similarly, the NIC population showed comparable ancestry proportions, comprising 40.27% EUR, 13.70% AFR, and 46.04% NAT (Figure 1).

**Figure 2:**
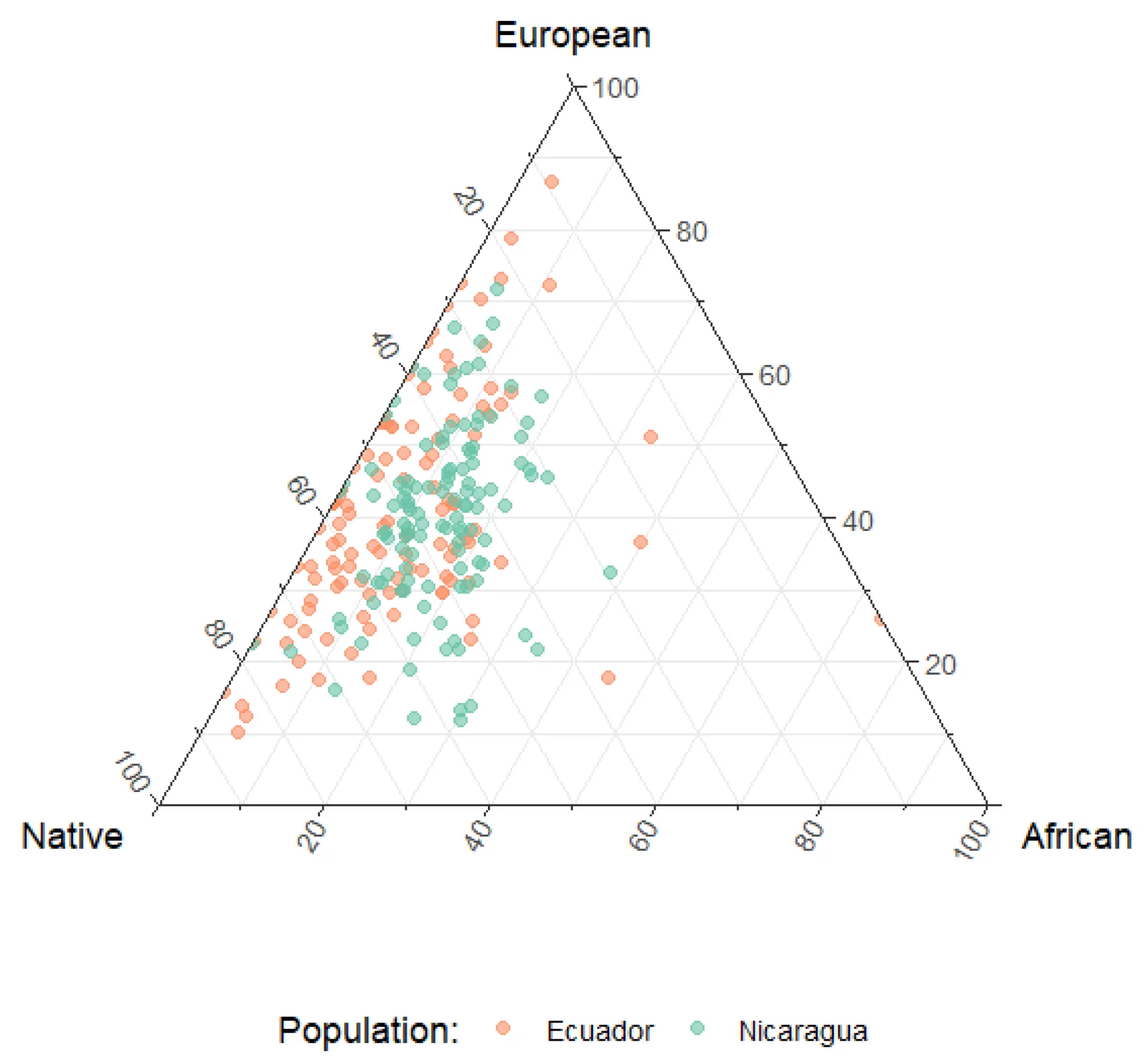
Proportion of Native American, African, and European ancestry in the combined Ecuadorian and Nicaraguan populations (n = 236 healthy volunteers).

When both populations were analyzed jointly, the overall genomic ancestry composition was 40.40% EUR, 11.40% AFR, and 48.16% NAT (Figure 2).

### 2.2. Association Between Genomic Ancestry and CYP450 Genetic Variability

#### 2.2.1. Association Between Genomic Ancestry and Allele Frequencies

Genotype distributions for *CYP2D6*, *CYP2C9*, *CYP2C19*, *CYP1A2*, and *CYP3A4* were consistent with Hardy–Weinberg equilibrium (*p* > 0.05), indicating that the observed genotype frequencies were compatible with those expected under HWE assumptions (Supplementary Materials; Tables S2 and S3).

No significant associations were identified between genomic ancestry and allele distribution for *CYP1A2* or *CYP3A4* (Table 1).

In contrast, Table 2 shows a significant positive association between the reduced-function allele CYP2C9*2 and the European (EUR) ancestry component (β = 0.331, adjusted-*p* = 0.049), indicating that individuals with a greater proportion of European ancestry are more likely to carry this variant. Regarding *CYP2C19*, the loss-of-function allele CYP2C19*2 was significantly associated with EUR ancestry (β = 0.470, adjusted-*p* = 0.029), indicating an increased prevalence of this variant among individuals with a higher European ancestral component.

Regarding *CYP2D6* variants, the AFR ancestry component showed a significant positive association with CYP2D6*17 alleles (β = 0.82, *adjusted-p* = 0.001). A positive association was also observed for CYP2D6*4 (β = 0.61, nominal-*p* = 0.048), although this association did not remain statistically significant after FDR correction (*adjusted-p* = 0.132). In addition, EUR ancestry was positively associated with CYP2D6*41 (β = 0.33, *adjusted-p* = 0.01). NAT ancestry showed a nominal positive association with the wild-type allele CYP2D6*1 (β = 0.80, adjusted-*p* = 0.034), which was not retained after multiple-testing correction (*adjusted-p* = 0.113) (Table 3).

#### 2.2.2. Association Between Genomic Ancestry and Genotype-Predicted Metabolizer Groups

Genotype-predicted metabolizer groups: poor metabolizer (gPM), intermediate metabolizer (gIM), normal metabolizer (gNM), rapid metabolizer (gRM) and ultrarapid metabolizer (gUM) were derived for *CYP3A4*, *CYP2C9*, *CYP2C19* and *CYP2D6* according to established genotype-to-phenotype translation recommendations previously described [11,12,13]. The corresponding allele function assignments and phenotype translation criteria are summarized in Supplementary Materials, Table S4. The association between genomic ancestry and predicted metabolizer groups was explored using logistic regression (Table 4).

For *CYP3A4*, no significant associations were identified between *CYP3A4*-predicted metabolizer groups and genomic ancestry. For *CYP2C9*, EUR ancestry was positively associated with the gIM phenotype (β = 2.74, adjusted-*p* = 0.033) and negatively associated with the gNM phenotype (β = −2.76, *p* = 0.033). Regarding *CYP2C19*, a negative association was observed between EUR ancestry and the gNM phenotype (β = −2.41, nominal-*p* = 0.017), although this association did not remain statistically significant after FDR correction (adjusted-*p* = 0.068). Finally, for *CYP2D6*, AFR ancestry was positively associated with the gIM (β = 3.53, adjusted-*p* = 0.0047) and the gPM (β = 5.631, adjusted-*p* = 0.026). In contrast, the gNM was negatively associated with AFR ancestry (β = −5.75, adjusted-*p* = 0.004).

### 2.3. Correlation Between Genomic Ancestry and Measured CYP450 Enzyme Capacity, as Measured by logMR

**Figure 3:**
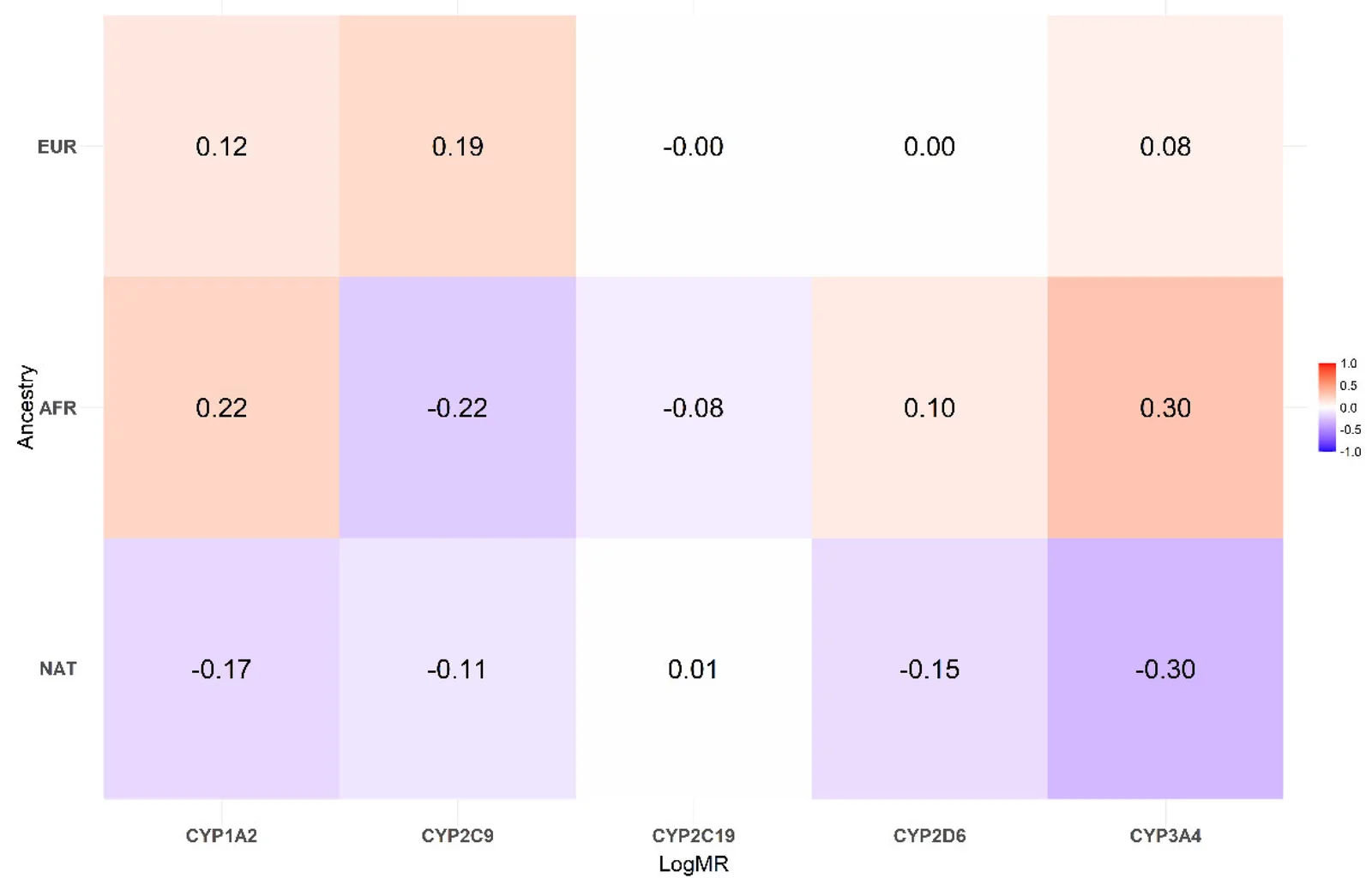
Correlation heatmap between genomic ancestry components (EUR: European; NAT: Native American; AFR: African) and logMR values for each enzyme (CYP1A2, CYP2C9, CYP2C19, CYP2D6, and CYP3A4).

The relationship between measured enzymatic activity, expressed as the logarithm of the metabolic ratio (logMR), for each CYP enzyme (CYP1A2, CYP3A4, CYP2C9, CYP2C19, and CYP2D6) and genomic ancestry was evaluated (Figure 3). For *CYP3A4*, higher logMR values, indicating lower enzymatic activity, showed a moderate positive correlation with AFR ancestry (r = 0.30, *p* = 0.001), whereas a moderate negative correlation was observed with NAT ancestry (r = −0.30, *p* = 0.001), representing the strongest ancestry–enzyme metabolic capacity relationships observed. A similar, although weaker, pattern was observed for *CYP1A2*, with a significant positive correlation with AFR ancestry (r = 0.22, *p* = 0.020), whereas the correlation with NAT ancestry was weak and did not reach statistical significance (r = −0.17, *p* = 0.076). No significant correlation was observed with EUR ancestry (r = 0.12, *p* = 0.203). In contrast, *CYP2C9* showed an opposite pattern, with logMR positively correlated with EUR ancestry (r = 0.19, *p* = 0.049) and negatively correlated with AFR ancestry (r = −0.22, *p* = 0.023). For *CYP2D6*, only very weak correlations were observed between logMR and AFR ancestry (r = 0.10, *p* = 0.281) and NAT ancestry (r = −0.15, *p* = 0.111), neither of which reached statistical significance. Regarding *CYP2C19*, only weak correlations were observed, with no statistically significant relationships for NAT or EUR ancestry.

Overall, no significant associations have been observed to suggest that ancestry plays a decisive role in the variability of metabolic activity.

### 2.4. Relationship Between Genotype-Predicted Metabolizer Groups and Measured Metabolic Capacity

**Figure 4:**
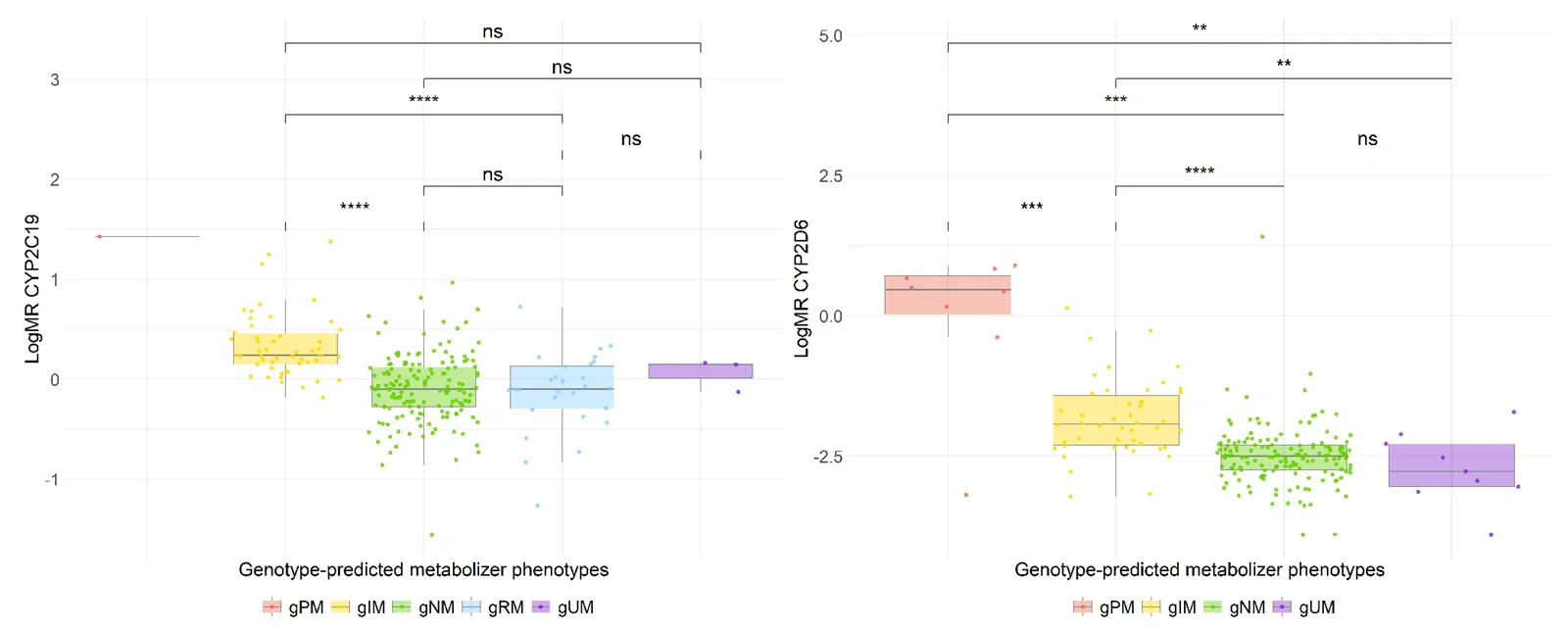
Relationship between logMR (measured metabolizer phenotype with a test-drug, CEIBA cocktail) and genotype-predicted metabolizer groups for *CYP2C19* and *CYP2D6*. Statistical significance for pairwise comparisons was assessed using the Wilcoxon rank-sum test. Statistical significance: **** (*p* < 0.0001); *** (*p* < 0.001); ** (*p* < 0.01); ns: not significant; gPM: poor metabolizer; gIM: intermediate metabolizer; gNM: normal metabolizer; gRM: rapid metabolizer; gUM: ultrarapid metabolizer.

The logMR differed significantly among metabolizer groups predicted from *CYP2C19* and *CYP2D6* genotypes. Specifically, differences in dextromethorphan (DM) logMR were observed among most genotype-predicted metabolizer groups (gPM, gIM, gNM, and gUM), with higher logMR values associated with lower CYP2D6 metabolic capacity. However, considerable overlap in logMR distribution was observed between groups (Figure 4).

For *CYP2C19*, significant differences in omeprazole logMR were observed among the gIM, gNM, and gRM groups (*p* < 0.0001), whereas no significant differences were detected between gPM and the other metabolizer categories (Figure 4).

**Figure 5:**
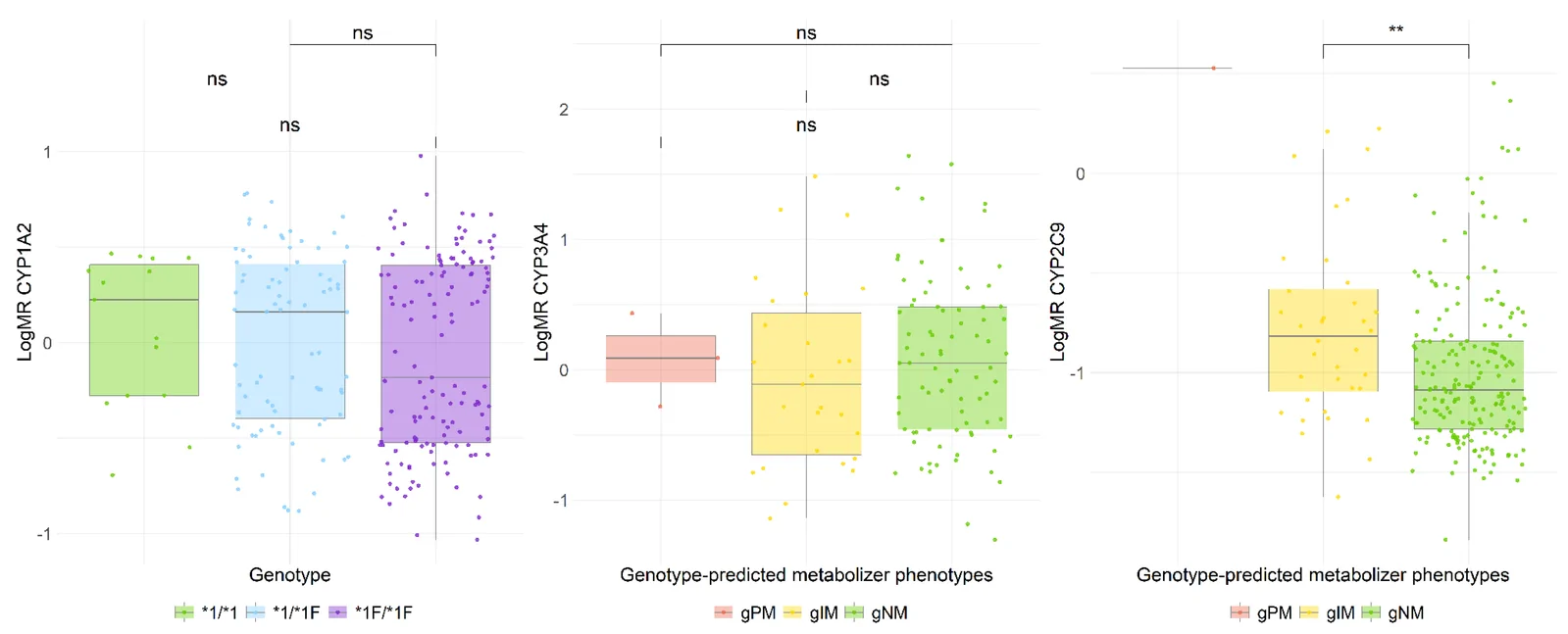
Relationship between *CYP1A2*, *CYP3A4*, and *CYP2C9* genotype-predicted metabolizer groups and logMR. Statistical significance for pairwise comparisons was assessed using the Wilcoxon rank-sum test ** Statistical significance (*p* < 0.01); ns: not significant; gPM: poor metabolizer; gIM: intermediate metabolizer; gNM: normal metabolizer.

Regarding CYP2C9, differences in losartan logMR were only observed between gIM and gNM (*p* = 0.003), although the number of gPM individuals was limited (Figure 5).

No significant differences were observed across genotype predicted metabolizer groups for CYP1A2 and CYP3A4 (Figure 5).

Overall, while some statistically significant differences were identified, substantial overlap in measured metabolic activity was observed among genotype-predicted groups across all genes.

## 3. Discussion

Populations in Latin America, such as those in ECU and NIC, are characterized by a complex history of admixture [14]. This historical process has resulted in contemporary populations with substantial EUR and NAT ancestral components, as well as AFR ancestry derived from forced migration and population movements from various regions of Africa [1,2]. Within this context, the primary aim of the present study was to evaluate the contribution of genomic ancestry to pharmacogenetic variability in ECU and NIC populations, focusing on ancestry-related differences in allele frequencies, genotype-predicted metabolizer groups, and measured metabolic activity (logMR). As a secondary objective, and after establishing that genomic ancestry alone does not reliably predict metabolic phenotype, the relationship between genotype-predicted metabolizer categories and measured enzymatic activity was evaluated.

This complementary analysis provides functional assessment of genotype-based phenotype predictions and helps determine their accuracy in highly admixed populations. Furthermore, this framework facilitates the subsequent investigation of the relationship between the measured metabolic phenotype, expressed as logMR, and each ancestral component.

Despite the considerable geographical distance (NIC is located in central and ECU in south America) between these two countries, the genomic ancestry analyses revealed comparable proportions of EUR, NAT, and AFR ancestry components, with an average composition of 40.40% EUR, 48.16% NAT, and 11.40% AFR (Figure 2). These findings are consistent with previous studies describing these populations, in which EUR and NAT components are typically the predominant ancestral contributions, often present in nearly equivalent proportions [2,6,10]. However, population-specific studies have also reported different ancestry distributions depending on the analytical approach and the geographic origin of sampled individuals [15]. For instance, it has been concluded that the NIC population is unmistakably tri-hybrid, deriving its genetic ancestry from EUR (46.9%), NAT (36.8%), and AFR (16.3%) populations [10]. This similarity is noteworthy given the distinct geographic and historical backgrounds of both populations and likely reflects the shared admixture processes that have shaped many Latin American populations. However, similar global ancestry proportions do not necessarily imply identical demographic histories or population genetic structures.

Although the ECU and NIC populations showed similar proportions of ancestral components, their underlying genetic structure may still differ. NAT ancestry is not homogeneous across the Americas, and Native populations from different geographic regions exhibit distinct patterns of allele frequencies [16]. For example, NAT populations from Central America showed a higher frequency of *CYP2D6* gPMs (10.20%) compared with populations from South America, and the CYP2D6*4 allele has a difference in the [17] frequency in South Native Americans (4.44%) when compared with North NAT (22.45%) [15]. In this RIBEF-CEIBA population study, one of the largest pharmacogenomic studies conducted in Latin America (n = 6060 individuals), substantial interethnic differences in *CYP2D6*, *CYP2C9*, and *CYP2C19* variants were identified. Native Americans exhibited distinct allele and predicted metabolizer phenotype frequencies compared with other groups [17]. These differences between NAT populations are also present within South American populations from the Andean highlands, which show a lower frequency of the *VKORC1* rs9923231. T allele compared with populations from the Amazonian lowlanders [18]. These findings suggest that differences in local genetic background may contribute to variability in pharmacogenetic profiles, even among populations with similar global ancestry proportions, underscoring the need for population-specific pharmacogenomic strategies and greater inclusion of underrepresented populations in precision medicine research.

Regarding the frequency distribution of pharmacogenetic alleles, the associations observed in the present study are consistent with the known global distribution of CYP variants. The CYP2C9*2 and CYP2D6*41 alleles occur at relatively high frequencies in EUR populations (13.18% and 9.71%, respectively) and Middle East and North Africa (12.05% and 15.07% respectively). In contrast, these alleles are much less frequent in East Asians populations (0.016% and 2.25%, respectively) and in Sub-Saharan African populations (2.13% and 2.59%, respectively) [17,19]. Consequently, populations with higher EUR ancestry are expected to show increased frequencies of these variants compared with populations enriched for East Asian or Sub-Saharan AFR ancestry.

NAT populations exhibit lower frequencies of the CYP2C9*2 allele than other groups studied, including individuals from the Iberian Peninsula, White Latin Americans, and Afro-descendant populations. Likewise, CYP2D6*41 shows variation in allele distribution across populations, with reported frequencies of 0.92% in NAT populations, 6.74% in White Latin Americans, and 8.89% in Iberian populations as shown in RIBEF-CEIBA study [17].

In contrast, the CYP2D6*17 allele has been consistently reported at a high frequency, in Sub-Saharan AFR populations (18.77%), whereas it is virtually absent in EUR and East Asian populations (0%). These population differences support the positive association observed between the CYP2D6*17 allele and the AFR ancestral component [19]. Similarly, the CYP2C19*17 allele was more frequent in admixed Latin American (12.33%), Afro-Latin American (21.34%), Iberian (19.89%), and White Latin American (16.10%) populations, whereas it was rare in NAT populations (1.59%) across the Americas, as reported in a previous RIBEF-CEIBA study [17]. In addition, frequencies of approximately 1% in East Asian populations and 22.14% in EUR populations for the same allele has been reported [19].

Finally, the frequency of the CYP2D6*4 allele observed (11.09%) (Supplementary Table S2) is higher than even that reported in some recent studies conducted in AFR populations, where the frequency ranges from 1 to 3% [20,21].

To associate the frequency alleles with genomic ancestry, the present findings are generally consistent with those reported by previous studies [6], particularly the positive associations between EUR ancestry and the CYP2D6*41 and CYP2C9*2 alleles, as well as the positive association between AFR ancestry and CYP2D6*17 (Table 1, Table 2 and Table 3) [22]. The CYP2C9*2 findings are particularly relevant to the work of the RIBEF Network, as this was one of the studies that laid the groundwork for the line of research it has pursued over the past 20 years [6,17].

However, some discrepancies were observed in the present study, such as a nominal positive association between CYP2D6*4 and AFR ancestry (Table 3), although it did not remain statistically significant after FDR correction. This exploratory finding contrasts with previous reports linking this allele predominantly with EUR ancestry in the RIBEF-CEIBA population study [6]. In addition, the CYP2C19*2 allele showed a positive association with the EUR ancestral component, and the CYP2C19*17 allele showed a negative association with NAT ancestry in [6]. These differences may reflect population-specific genetic drift, sampling variability, or the complex recombination patterns present in admixed populations.

Overall, these findings confirm that genomic ancestry contributes to shaping the distribution of pharmacogenetic relevant variants. Nevertheless, the clinical impact of these genetic differences cannot be inferred solely from allele frequencies and requires functional evaluation of their effects on drug-metabolizing phenotypes. These ancestry-related allele patterns were partly reflected in the analysis of genotype-predicted metabolizer groups. For *CYP2C9*, greater EUR ancestry was associated with a higher prevalence of the gIM and a lower prevalence of the gNM. This finding is consistent with the positive association observed between EUR ancestry and the decreased-function CYP2C9*2 and CYP2C19*2 allele, which may contribute to a shift from the gNM to the gIM [23,24]. Similarly, for *CYP2D6*, greater AFR ancestry was associated with a higher prevalence of gIM and gPM and a lower prevalence of gNM. These results are consistent with the positive associations identified between AFR ancestry and CYP2D6*17 alleles [25]. CYP2D6*4 is a no-function allele and CYP2D6*17 confers decreased function, their greater representation may partly explain the higher prevalence of gIM and gPM among individuals with higher AFR ancestry. It is important to consider that genotype-to-phenotype translation models used in this study were originally developed in non-admixed populations, and their predictive accuracy may be reduced in highly admixed groups such as those from ECU and NIC.

In agreement with previous findings, the analysis of the relationship between genomic ancestry and measured metabolic activity (metabolic phenotype) revealed that AFR ancestry tended to be associated with higher logMR values (indicative of lower enzymatic activity) for CYP1A2 and CYP3A4, whereas NAT ancestry showed the opposite trend. In contrast CYP2C9 displayed a different pattern, with higher logMR correlated with EUR ancestry (Figure 3) [26,27,28].

Despite these trends, the strength of the correlations was weak to moderate (r between 0.10 and 0.30), indicating that ancestry alone explains just a limited proportion of the variability in metabolic phenotype. These results suggest that genomic ancestry may act as a contributing factor but is insufficient as a standalone predictor of intrinsic enzyme activity.

Taken together, these findings support a hierarchical relationship in which genomic ancestry contributes to the distribution of pharmacogenetic variants and genotype-predicted metabolizer categories, whereas its association with experimentally measured metabolic activity is considerably weaker.

The comparison of genotype-predicted metabolizer groups with measured metabolic activity constituted a complementary component of the study. Although statistically significant differences in logMR were observed between some genotype-predicted metabolizer groups, particularly for *CYP2D6* and *CYP2C19*, the overall distributions showed substantial overlap between categories (Figure 4 and Figure 5). This overlap indicates that genotype-based phenotype prediction does not fully capture the variability in enzymatic activity observed in these populations. Accordingly, neither genomic ancestry nor genotype-predicted metabolizer status alone was sufficient to accurately predict individual CYP450 metabolic activity. In several cases, individuals classified into different genotype-predicted metabolizer categories exhibited similar metabolic activity, and conversely, individuals within the same genotype category showed widely varying logMR values. These findings are consistent with previous studies conducted in ECU and NIC populations, which have reported significant genotype–phenotype discordance for CYP450 enzymes [26,27,28].

A specific methodological consideration applies to the interpretation of the two dextromethorphan-derived phenotyping indices. Although the formation of 3-metoxy-morphinan (3MM) is mediated predominantly by CYP3A4/5 and therefore provides a mechanistic basis for using the DM/3MM ratio as an index of CYP3A activity, this ratio is not completely independent of CYP2D6 activity. Previous in vivo studies have therefore concluded that the DM/dextrorphan (DX) and DM/3MM ratios are not strictly independent [29,30,31]. This metabolic interdependence is particularly relevant in the present study because CYP2D6 genetic variation is one of the principal variables investigated. Part of the variability attributed to the CYP3A-related logMR could consequently reflect CYP2D6-dependent differences in substrate availability or secondary 3MM metabolism. Thus, the observed DM/3MM logMR should be interpreted as a functional index of predominantly CYP3A-mediated N-demethylation under the standardized CEIBA protocol, rather than as an isolated or exclusively CYP3A4-specific phenotype. This limitation may also contribute to the absence of clear differences among CYP3A4 genotype-predicted groups and to the overlap observed in Figure 5.

Taken together, the present results reinforce the evidence that genotype-predicted metabolizer classifications do not fully capture the interindividual variability observed in measured metabolic activity. Similar findings have previously been reported in the populations studied here (ECU and NIC) [32,33] and in Mexico [34], highlighting the contribution of additional genetic and non-genetic factors to CYP450 metabolic phenotypes.

The combined analysis of ancestry, genotype, and measured phenotype highlights the complexity of pharmacogenetic variability in admixed populations. However, it should be noted that the present study evaluated genomic ancestry estimated from ancestry-informative markers and did not assess self-identified ethnicity or race. Therefore, the findings should be interpreted within the context of genetic ancestry rather than social or cultural classifications. Consistent with this distinction, studies in Brazilian populations have shown that genomic ancestry, self-reported color, and geographic origin capture related but non-equivalent dimensions of pharmacogenomic diversity [35].

While genomic ancestry is associated with allele frequencies and contributes modestly to metabolic variability, neither ancestry nor genotype alone adequately explains the observed phenotypic diversity. In this context, genomic ancestry may act as an integrative proxy capturing part of the variability not explained by genotyping alone. However, the modest strength of ancestry–phenotype associations observed in this study indicates that its predictive value remains limited and rather than confirming the robustness of genotype-based prediction models, the present results underscore their limitations and suggest that additional factors must be incorporated into pharmacogenetic frameworks. These findings also support the need for population-specific evaluation of pharmacogenetic markers, particularly in underrepresented and admixed Latin-American populations.

From a clinical perspective, these results have important implications for the implementation of personalized medicine in Latin America. The limited concordance between genotype-predicted and measured phenotypes suggests that reliance solely on genotyping may lead to suboptimal prediction of drug metabolism in these populations [32,33,34].

The use of phenotyping approaches with a test-drug, such as the CEIBA cocktail, may provide complementary information and improve the accuracy of metabolic assessment, particularly in contexts where multiple non-genetic factors influence intrinsic enzyme activity.

From a methodological standpoint, the findings highlight the importance of integrating functional phenotyping into pharmacogenetic studies, especially when working with admixed populations. They also emphasize the need for careful interpretation of genotype–phenotype relationships and caution against overgeneralization of models derived from other populations.

Several limitations should also be acknowledged. First, the sample size, particularly within specific genotype-predicted metabolizer categories (e.g., gPM or gUM), was limited, which may reduce statistical power and the ability to detect differences. Consequently, associations observed for these low-frequency categories should be interpreted with caution. Second, although several exclusion criteria and pre-phenotyping restrictions were applied to minimize the influence of known environmental factors on CYP450 activity, oral contraceptives, additional determinants such as smoking, alcohol consumption, BMI, dietary patterns, and other environmental exposures were not evaluated in the present analyses. Therefore, residual confounding cannot be completely excluded when interpreting the observed associations between genomic ancestry and metabolic phenotypes. Third genotype-to-phenotype translation models used in this study were originally developed in non-admixed populations, and their predictive accuracy may be reduced in highly admixed groups such as those from ECU and NIC. Finally, phenotyping using the CEIBA cocktail reflects metabolic activity at a single time point and may be influenced by transient physiological or environmental factors, limiting its ability to fully represent long-term metabolic capacity. In addition, because DM was used as a common probe for CYP2D6 and CYP3A4, the corresponding MRs cannot be regarded as fully independent. CYP2D6 genetic and functional variation may influence the DM/3MM ratio through competition for the parent substrate and further metabolism of 3MM. The CYP3A4-related findings should therefore be interpreted cautiously and should ideally be confirmed using a more selective CYP3A4 probe or by analyses explicitly adjusted for CYP2D6 activity.

In conclusion, genomic ancestry contributes to the distribution of CYP450 pharmacogenetic variants and genotype-predicted metabolizer categories in Ecuadorian and Nicaraguan populations. However, the observed ancestry–phenotype associations were modest, and substantial overlap was observed across genotype-predicted metabolizer groups. These findings indicate that neither genomic ancestry nor current genotype-to-phenotype translation systems fully explain individual CYP450 metabolic variability, highlighting the complexity of pharmacogenetic phenotype prediction in admixed populations.

These findings highlight the limitations of conventional pharmacogenetic prediction models in admixed populations and support the need for integrative approaches combining genotyping, phenotyping, and population-specific data to improve the implementation of precision medicine in Latin America. This study is aligned with the need to include genomic ancestry and genetic diversity in clinical research, which is the main motivation behind the RIBEF Network’s study and the basis for the Merida/T’Ho Declaration issued by this Network together with Council of International Organizations of Medical Sciences (CIOMS) in 2020 [36].

## 4. Materials and Methods

### 4.1. Inclusion and Exclusion Criteria

A total of 236 healthy volunteers were studied from various regions of NIC (N = 118), located in the center of Central America [32], and from ECU (N = 118) [33], situated in the Andean region of South America. Of these volunteers the mean age was 22.8 (SD ± 2.02) years in ECU and 20.8 (SD ± 3.34) years in NIC, with women representing 54.24% and 51.69% of each cohort, respectively. Overall, 52.97% of the participants were women, with a mean age of 21 years (SD ± 2.94). Health status was verified through standard clinical assessments and routine laboratory analyses to confirm that participants fulfilled the study inclusion criteria as healthy volunteers. A detailed review of each participant’s medical history was conducted before enrollment. Only unrelated individuals were included in the study population to ensure the independence of observations in genomic ancestry and pharmacogenetic analyses. Exclusion criteria comprised current pregnancy or suspicion thereof, a documented history of adverse drug reactions, the use of drugs or central nervous system stimulants, and any medication intake within the two weeks preceding participation. In the case of the Nicaraguan cohort, women using oral contraceptives were eligible for inclusion.

The study protocols received approval from the Ethics Committee for Biomedical Research (CEIB) “Dr. Uriel Guevara Guerrero” (FW00004523/IRB00003342) at the Universidad Nacional Autónoma de NIC (León, NIC), and from the Research Ethics Committee on Human Subjects of the Universidad San Francisco de Quito (Quito, ECU) under code 2014-002B-SUB. All procedures complied with the principles of the Declaration of Helsinki, and written informed consent was obtained from all participants.

### 4.2. Genomic Ancestry Analysis

For the ancestry analysis, a previously described panel of 83 ancestry-informative markers (AIMs) was used [37]. The original panel compromised 107 AIMs, which were selected to be adequately distributed throughout the genome and sufficiently distant from one another to be in linkage equilibrium in the ancestral populations, with an average distance between markers of approximately 2.4 × 10^7^ bp [37]. After exclusion of markers with missing genotype data in the previously generated dataset, 83 AIMs were retained for the present analysis. These markers were genotyped in all individuals, included in the study and in the comparative populations.

Spaniards (n = 114) and Native American individuals from Peru (n = 296) and Yoruba individuals (n = 119) from the 1000 Genomes Project [38] were included as comparative populations representing European-, Native American-, and African-related genetic backgrounds, respectively. The study and comparative populations were analyzed jointly using ADMIXTURE software (v1.3.0) [39] under an unsupervised framework, assuming three broad ancestral components (K = 3). Accordingly, the resulting EUR, NAT, and AFR components were interpreted as broad European-, Native American-, and African-related ancestry components rather than as direct estimates of ancestry from the specific contemporary comparative populations.

### 4.3. Genotype and Assignment of Genotype-Predicted Metabolizer Groups

Analysis of CYP450 allelic variants (Supplementary Table S1) was performed using commercially available TaqMan^®^ gene expression assays (Applied Biosystems, Foster City, CA, USA). PCR plates were analyzed using the ABI 7300 real-time PCR system (Applied Biosystems, Foster City, CA, USA) following the methodology described in previous articles [32]. *CYP2D6* gene duplications and the CYP2D6*5 gene deletion were determined using XL-PCR, as previously described [40].

Not all study participants were included in all genotyping analyses. Consequently, the number of individuals contributing genotype data differed among genes, corresponding to 235 individuals for *CYP1A2*, 233 for *CYP2C9*, 183 for *CYP3A4*, 221 for *CYP2D6* and 236 for *CYP2C19.* Therefore, allele frequencies were calculated using the subset of individuals for whom genotype data were available for each gene. To assign genotype-predicted metabolizer phenotypes, genotype-to-phenotype translation recommendations from CPIC and DPWG guidelines were applied according to the analyzed CYP450 variants [11,12,13].

### 4.4. Phenotyping Using CEIBA Cocktail

Enzymatic activity was evaluated using the validated CEIBA cocktail protocol, originally developed by the RIBEF network for pharmacogenetic research in indigenous and admixed populations across the Americas and described in previous studies [41]. This methodology employs single oral doses of specific probe substrates for *CYP2D6* and *CYP3A4* (dextromethorphan, 30 mg), *CYP2C9* (losartan, 25 mg), *CYP2C19* (omeprazole, 20 mg), and *CYP1A2* (caffeine, 100 mg) which were given to each participant. The metabolic ratio (MR) for each gene was defined as the ratio between the compound and its CYP isoform specific metabolite in plasma 4 h after dosing, and 3-h for DM related measurements [42]. Higher MR values indicate lower enzymatic activity [43]. For statistical analyses, metabolic ratio values were log-transformed using log10. Thus, MRs were defined as follows: dextromethorphan/dextrorphan (CYP2D6), omeprazole/5-hydroxyomeprazole (CYP2C19), losartan/E-3174 (CYP2C9), caffeine/paraxanthine (CYP1A2), and dextromethorphan/3-metoxy-morphinan (CYP3A4) [41].

DM is converted to DX through CYP2D6-mediated O-demethylation and to 3MM through N-demethylation, mediated predominantly by CYP3A4/5. Recombinant-enzyme and human liver microsome studies have estimated that CYP3A4 accounts for more than 90% of 3MM formation under the evaluated in vitro conditions, providing the mechanistic basis for using the DM/3MM ratio as an index of CYP3A-mediated N-demethylation [29,30,31]. However, because the CYP2D6 and CYP3A pathways share DM as the parent substrate and 3MM may undergo subsequent CYP2D6-mediated O-demethylation, the two DM-based ratios are not completely independent. Accordingly, the DM/3MM ratio was interpreted in this study as an index of predominantly CYP3A-mediated activity rather than as an exclusively CYP3A4-specific measurement [29,30,31].

To minimize confounding factors, subjects were instructed to abstain from consuming caffeine-containing products for 72 h before probe administration and throughout the testing period. They fasted for 12 h before drug intake and for an additional 4 h afterward. The collection of a single sample was previously validated [41]. Compliance was monitored to ensure avoidance of medications, herbal supplements, or dietary components known to modulate CYP intrinsic enzyme activity, following procedures previously established for NIC [32] and ECU [33] populations.

Blood samples were collected 4 h after dosing, centrifuged at 3500 rpm for 10 min, and plasma aliquots were stored at −20 °C until analysis by LC-MS/MS using a method previously validated [43]. Briefly, calibration curves were linear (R^2^ > 0.99), and intra- and inter-day precision (i.e., RSD values) and accuracy were calculated within ±15%. Matrix effects were evaluated and compensated using internal standards [43].

### 4.5. Statistical Analysis

#### 4.5.1. Distribution of Genotypes and Genotype-Predicted Metabolizer Groups

The frequencies of the *CYP1A2*, *CYP3A4*, *CYP2D6*, *CYP2C9*, and *CYP2C19* alleles were calculated for each population. In addition, the frequencies of the predicted metabolic phenotypes associated with *CYP3A4*, *CYP2D6*, *CYP2C9*, and *CYP2C19* were also determined for each population. Hardy–Weinberg equilibrium was tested for each allele in each population using the χ2 test with Yates’ correction [44].

#### 4.5.2. Associations of Genomic Ancestry with Allele Distributions and Genotype-Predicted Metabolizer Groups

Individual-level allele dosage for CYP1A2, CYP3A4, CYP2C9, CYP2C19, and CYP2D6 was coded as 0, 1, or 2 copies of the corresponding allele. Linear regression models were then used to evaluate the association between allele dosage and the proportions of EUR, AFR, and NAT ancestries, following the methodological approach previously described by Rodrigues-Soares et al. (2020) [6]. For each model, the regression beta coefficient (β), 95% confidence interval, and *p*-value were estimated. False discovery rate (FDR)-adjusted *p*-values were also calculated to account for multiple testing. All analyses were performed using the *lm* function in the R environment (version 4.4.3).

For genotype-predicted metabolizer groups (gPM, gIM, gNM, gRM, and gUM), logistic regression models were used to evaluate their association with the proportions of EUR, AFR, and NAT ancestries. Following the methodological approach described by Rodrigues-Soares et al. (2020) [6], each ancestry component was analyzed separately to avoid multicollinearity arising from the compositional nature of EUR, AFR, and NAT proportions. For each model, the regression beta coefficient (β), 95% confidence interval, and *p*-value were estimated. False discovery rate (FDR)-adjusted *p*-values were also calculated to account for multiple testing. The different analyses were performed using the glm function with a binomial distribution in the R environment.

#### 4.5.3. Relationship Between Genomic Ancestry and Metabolic Ratio

Spearman’s rank correlation analysis was used to assess the associations between individual ancestry proportions and logMR values for each CYP enzyme. All analyses were performed using the stats package in R software (version 4.4.3).

#### 4.5.4. Relationship Between Genotype-Predicted Metabolizer Groups and Measured Metabolic Capacity

Differences in logMR values between genotype-predicted metabolizer groups were assessed using pairwise Wilcoxon rank-sum tests. This non-parametric approach was selected because of the unequal sample sizes across metabolizer groups and the distribution of logMR values. All possible pairwise comparisons between metabolizer groups were evaluated. Boxplots were also generated to visualize the distribution of logMR values across genotype-predicted metabolizer groups, together with the corresponding pairwise comparisons. All statistical analyses and graphical representations were performed in the R environment (version 4.4.3).

## Figures and Tables

**Table 1:** Association of *CYP1A2* and *CYP3A4* alleles with population ancestry (n = 236 healthy volunteers). Univariate linear regression analysis: β coefficients (95% CI), nominal *p*-values, and FDR-adjusted *p*-values for each association. Numbers in parentheses indicate the allele count.

	*CYP1A2*		*CYP3A4*		
allele	*1 (112)	*1F (358)	*1 (305)	*1B (57)	*22 (4)
NAT ancestry					
β (95% CI)	−0.50 (−1.08, 0.09)	0.496 (−0.09, −1.08)	0.04 (−0.56, 0.64)	−0.01 (−0.59, 0.59)	−0.04 (−0.2, 0.12)
*p*-Value	0.097	0.097	0.890	0.993	0.635
Adjusted *p*-Value	0.097	0.097	0.993	0.993	0.993
AFR ancestry					
β (95% CI)	0.635 (−0.22, 1.49)	−0.635 (−1.49, 0.22)	−0.58 (−1.42, 0.26)	0.59 (−0.24, 1.42)	−0.01 (−0.24, 0.22)
*p*-Value	0.144	0.144	0.173	0.162	0.945
Adjusted *p*-Value	0.144	0.144	0.259	0.259	0.945
EUR ancestry					
β (95% CI)	0.185 (−0.39, 0.76)	−0.185 (−0.76, 0.39)	0.24 (−0.34, 0.82)	−0.28 (−0.85, 0.29)	0.04 (−0.12, 0.20)
*p*-Value	0.525	0.525	0.422	0.340	0.612
Adjusted *p*-Value	0.525	0.525	0.612	0.612	0.612

**Table 2:** Association of *CYP2C9* and *CYP2C19* alleles with population ancestry (n = 236 healthy volunteers). Univariate linear regression analysis: β coefficients (95% CI), nominal *p*-values, and FDR-adjusted *p*-values for each association. Numbers in parentheses indicate the allele count.

	*CYP2C9*			*CYP2C19*		
	*1 (428)	*2 (24)	*3 (14)	*1 (383)	*2 (52)	*17 (37)
NAT ancestry						
β (95% CI)	0.26 (−0.11, 0.62)	−0.28 (−0.57, 0.01)	0.02 (−0.22, 0.27)	0.51 (−0.01, 1.01)	−0.24 (−0.64, 0.17)	−0.27 (−0.66, 0.12)
*p*-Value	0.166	0.058	0.855	0.051	0.245	0.181
Adjusted *p*-Value	0.249	0.174	0.855	0.153	0.245	0.245
AFR ancestry						
β (95% CI)	0.32 (−0.22, 0.85)	−0.15 (−0.57, 0.28)	−0.17 (−0.53, 0.18)	0.64 (−0.10, 1.38)	−0.55 (−1.14, 0.04)	−0.09 (−0.66, 0.48)
*p*-Value	0.246	0.498	0.355	0.091	0.068	0.751
Adjusted *p*-Value	0.498	0.498	0.498	0.137	0.137	0.751
EUR ancestry						
β (95% CI)	−0.38 (−0.74, −0.03)	0.33 (0.05, 0.61)	0.05 (−0.19, 0.29)	−0.76 (−1.25, −0.27)	0.47 (0.08, 0.86)	0.29 (−0.09, 0.67)
*p*-Value	0.033	**0.021**	0.661	**0.002**	**0.019**	0.130
Adjusted *p*-Value	0.049	**0.049**	0.661	**0.006**	**0.029**	0.130

**Table 3:** Association of *CYP2D6* alleles with population ancestry (n = 236 healthy volunteers). Univariate linear regression analysis: β coefficients (95% CI), nominal *p*-values, and FDR-adjusted *p*-values for each association. Numbers in parentheses indicate the allele count.

	*CYP2D6*									
	*1 (244)	*2 (90)	*3 (2)	*4 (47)	*5 (9)	*10 (7)	*17 (13)	*29 (9)	*35 (10)	*41 (11)
NAT ancestry										
β (95% CI)	0.80 (0.06, 1.53)	0.18 (−0.39, 0.74)	0.03 (−0.06, 0.13)	−0.19 (−0.62, 0.24)	−0.02 (−0.23, 0.19)	0.03 (−0.14, 0.20)	−0.38 (−0.60, −0.16)	0.05 (−0.14, 0.24)	−0.08 (−0.28, 0.12)	−0.26 (−0.47, −0.06)
*p*-Value	**0.034**	0.541	0.463	0.380	0.839	0.720	**0.001**	0.581	0.454	**0.013**
Adjusted *p*-Value	0.113	0.726	0.726	0.726	0.839	0.800	**0.010**	0.726	0.726	0.065
AFR ancestry										
β (95% CI)	−1.52 (−2.58, −0.47)	−0.40 (−1.22, 0.41)	0.07 (−0.06, 0.20)	0.61 (0.01, 1.22)	0.30 (−0.01, 0.60)	0.01 (−0.24, 0.25)	0.82 (0.51, 1.13)	0.21 (−0.07, 0.48)	−0.19 (−0.48, 0.09)	−0.19 (−0.49, 0.11)
*p*-Value	**0.005**	0.329	0.298	**0.048**	0.053	0.966	**<0.001**	0.139	0.188	0.221
Adjusted *p*-Value	**0.025**	0.366	0.366	0.132	0.132	0.966	**0.001**	0.278	0.313	0.316
EUR ancestry										
β (95% CI)	−0.06 (−0.78, 0.67)	0.02 (−0.53, 0.57)	−0.06 (−0.15, 0.03)	−0.10 (−0.51, 0.31)	−0.11 (−0.32, 0.09)	−0.03 (−0.19, 0.13)	−0.02 (−0.24, 0.20)	−0.14 (−0.32, 0.04)	0.16 (−0.03, 0.35)	0.33 (0.13, 0.53)
*p*-Value	0.881	0.947	0.158	0.634	0.271	0.708	0.861	0.126	0.107	**0.001**
Adjusted *p*-Value	0.947	0.947	0.395	0.947	0.542	0.947	0.947	0.395	0.395	**0.010**

**Table 4:** Association between genomic ancestry and genotype-predicted metabolizer groups of *CYP3A4*, *CYP2C9*, *CYP2C19* and *CYP2D6.* Logistic regression analysis. Abbreviations: n: number of subjects, ND: No defined; β: Beta coefficient; CI: Confidence interval; AFR: African; EUR: European; NAT: Native American ancestry component.

	*CYP3A4*								
	NAT			AFR			EUR		
	β (95% CI)	*p*-value	*Adjusted p-value*	β (95% CI)	*p*–value	*Adjusted p-value*	β (95% CI)	*p*-value	*Adjusted p-value*
gPM (6)	0.08 (−5.98, 6.30)	0.979	0.979	2.69 (−5.64, 8.46)	0.422	0.422	−1.84 (−8.38, 4.15)	0.562	0.609
gIM (49)	−0.24 (−2.75, 2.27)	0.85	0.979	1.70 (−1.74, 5.14)	0.32	0.422	−0.63 (−3.11, 1.78)	0.609	0.609
gNM (128)	0.21 (−2.21, 2.64)	0.863	0.979	−2.12 (−5.55, 1.19)	0.208	0.422	0.86 (−1.47, 3.26)	0.473	0.609
	*CYP2C9*								
	NAT			AFR			EUR		
	β (95% CI)	*p*-value	*Adjusted p-value*	β (95% CI)	*p*-value	*Adjusted p-value*	β (95% CI)	*p* -value	*Adjusted p-value*
gPM (1)	ND	ND	ND	ND	ND	ND	ND	ND	ND
gIM (36)	−1.98 (−4.68, 0.66)	0.145	0.149	−2.47 (−7.21, 1.62)	0.274	0.274	2.76 (0.23, 5.35)	0.033	0.033
gNM (196)	1.94 (−0.68, 4.61)	0.149	0.149	2.58 (−1.49, 7.28)	0.250	0.274	−2.76 (−5.32, −0.25)	0.032	0.033
	*CYP2C19*								
	NAT			AFR			EUR		
	β (95% CI)	*p*-value	*Adjusted p-value*	β (95% CI)	*p*-value	*Adjusted p-value*	β (95% CI)	*p*-value	*Adjusted p-value*
gPM (1)	ND	ND	ND	ND	ND	ND	ND	ND	ND
gIM (50)	−0.35 (−2.69, 1.98)	0.768	0.768	−3.37 (−7.61,0.40)	0.1	0.260	1.57 (−0.67, 3.83)	0.169	0.225
gNM (153)	1.48 (−0.53, 3.53)	0.152	0.366	2.48 (−0.59, 5.84)	0.130	0.260	−2.41 (−4.24, −0.45)	0.017	0.068
gRM (28)	−0.97 (−3.93, 1.97)	0.518	0.691	0.73 (−3.73, 4.55)	0.726	0.726	0.57 (−2.31, 3.37)	0.694	0.694
gUM (4)	−4.73 (−11.6, 2.46)	0.183	0.366	−7.58 (−25.6, 4.73)	0.331	0.441	6.23 (−0.48, 13.2)	0.065	0.130
	*CYP2D6*								
	NAT			AFR			EUR		
	β (95% CI)	*p*-value	*Adjusted p-value*	β (95% CI)	*p*-value	*Adjusted p-value*	β (95% CI)	*p*-value	*Adjusted p-value*
gPM (8)	−0.40 (−5.61, 4.93)	0.883	0.883	5.63 (0.26, 10.9)	0.026	0.047	−4.50 (−10.7, 0.98)	0.126	0.504
gIM (46)	−1.53 (−4.00, 0.91)	0.221	0.442	3.53 (0.28, 6.95)	0.035	0.047	−0.37 (−2.78, 1.97)	0.757	0.757
gNM (167)	1.60 (−0.59, 3.84)	0.155	0.442	−5.75 (−9.33, −2.49)	0.001	0.004	1.14 (−0.98, 3.33)	0.297	0.594
gUM (9)	−1.55 (−6.44, 3.46)	0.540	0.720	3.50 (−2.67, 8.47)	0.188	0.188	−0.79 (−5.94, 3.98)	0.755	0.757

## Data Availability

The original contributions presented in this study are included in the article/Supplementary Material. Further inquiries can be directed to the corresponding authors.

## Supplementary Materials

The following supporting information can be downloaded at https://www.mdpi.com/article/10.3390/ph19091432/s1, Table S1: Cytochrome P450 allelic variants and Taqman^®^ assay utilized by genotyping by Real Time- PCR.; Table S2: *CYP1A2*, *CYP3A4*, *CYP2C9*, *CYP2C19* and *CYP2D6* allele frequencies; Table S3: *CYP1A2*, *CYP3A4*, *CYP2C9*, *CYP2C19* and *CYP2D6* genotype frequencies; Table S4: Genotype-predicter metabolizers groups frequency.

## Abbreviations

The following abbreviations are used in this manuscript:

CYP450	Cytochrome P450 enzyme
ECU	Ecuador
NIC	Nicaragua
EUR	European
NAT	Native American
AFR	African
gPM	Poor metabolizer
gIM	Intermediate metabolizer
gNM	Normal metabolizer
gUM	Ultrarapid metabolizer
gRM	Rapid metabolizer
logMR	Logarithm of the metabolic ratio

## Appendix A. Consortium of Authors: RIBEF-IBEROFEN Consortium for the Study of Phenotype–Genotype Relationships and Ancestry in Native American Populations

Adrián LLerena ^1,2,3,4^, Carla González de la Cruz ^1,2,3^, Carmen Mata-Martín ^1,2,4^, Juan Antonio Villatoro-García ^1,2,3^, Eva Peñas-Lledó ^1,2,3^, Pedro Dorado ^1,2,3^, Fernando-de-Andrés ^1,5^, Fernanda Rodrigues-Soares ^1,6^, Martha Sosa-Macías ^1,7^, Carlos Galaviz-Hernández ^1,7^, Enrique Terán ^1,8^, Catalina Altamirano-Tinoco ^1,9^, Ronald Ramírez-Roa ^1,10^.

1. RIBEF/SIFF Red y Sociedad Iberoamericana de Farmacogenética y Farmacogenómica, Spain.
2. INUBE University Institute for Bio-Sanitary Research of Extremadura. Badajoz, Spain.
3. Faculty of Medicine and Health Sciences, University of Extremadura. Badajoz, Spain.
4. Unit of Pharmacogenomics and Personalized Medicine. Clinical Pharmacology Service. Badajoz University Hospital, Extremadura Health Service (SES). Badajoz, Spain.
5. Department of Analytical Chemistry and Food Technology. Faculty of Pharmacy, University of Castilla-La Mancha. Albacete, Spain.
6. Department of Pathology, Genetic and Evolution, Universidade Federal do Triângulo Mineiro, Uberaba, Brazil.
7. IPN Instituto Politécnico Nacional. CIIDIR Unidad Durango. Academia de Genómica. Durango, México.
8. Colegio de Ciencias de la Salud, Universidad San Francisco de Quito, Quito, Ecuador.
9. Facultad de Ciencias Médicas, Universidad Nacional Autónoma de Nicaragua-León (UNAN-León), León, Nicaragua.
10. Facultad de Odontología, Universidad Americana, Managua, Nicaragua.
