# Supplementary material for: Association of Native American, European, and African Ancestry with CYP450 Pharmacogenetics and Metabolic Phenotypes in Admixed Latin American Populations from Ecuador and Nicaragua
Source: Pharmaceuticals (Basel). 2026 Sep 10;19(9):1432. doi: 10.3390/ph19091432 (PMC13610334; doi:10.3390/ph19091432)
Supplement: Supplementary file 1 [file pharmaceuticals-19-01432-s001.zip › pharmaceuticals-4504294-supplementary.pdf]

Table S1. Cytochrome P450 allelic variants and Taqman® assay utilized by genotyping by Real Time-PCR.

CYP Gene	CYP alleles	rs ID	Nucleotide change	Allele function	TaqMan® assay ID
	**1F*	rs762551	32035C>A	Higher inducibility	C_8881221_40
	**1K*	rs12720461	31469C>T	Decreased	C__30634146_10
CYP2C9	**2*	rs1799853	3608C>T	Decreased	C__25625805_10
	**3*	rs1057910	42614A>C	Decreased	C__27104892_10
	**6*	rs9332131	10601delA	None	C__32287221_20
CYP2C19	**2*	rs4244285	19154G>A	None	C_25986767_70
	**3*	rs4986893	17948G>A	None	C_27861809_10
	**4*	rs28399504	1A>G	None	C_30634136_10
	**5*	rs56337013	90033C>T	None	C_27861810_10
	**17*	rs12248560	−806C>T	Increased	C____469857_10
CYP2D6	**2*	rs1080985	−1584C>G	Normal	C_32407252_30
	**3*	rs35742686	2550 delA	None	C_32407232_50
	**4*	rs3892097	1847G>A	None	C__27102431_D0
	**6*	rs5030655	1708T>del	None	C_32407243_20
	**10*	rs1065852	100C>T	Decreased	C_11484460_40
	**17*	rs28371706	1022C>T	Decreased	C___2222771_40
	**29*	rs59421388	3184G>A	Decreased	C__34816113_20
	**35*	rs769258	31G>A	Normal	C__27102444_80
	**41*	rs28371725	2989G>A	Decreased	C_34816116_20
CYP3A4	**1B*	rs2740574	−392A>G	-	C___1837671_50
	*22**	rs35599367	15389C>T	Decreased	C__59013445_10

Table S2. *CYP1A2, CYP3A4, CYP2C9, CYP2C19* and *CYP2D6* allele frequency.

CYP Gene	CYP Alleles	N	Frequency (%)
CYP1A2	**1*	112	23.83
	**1F*	358	76.17
CYP3A4	**1*	305	83.33
	**1B*	57	15.57
	**22*	4	1.09
CYP2C9	**1*	428	91.85
	**2*	24	5.15
	**3*	14	3
CYP2C19	**1*	383	81.14
	**2*	52	11.02
	**17*	37	7.84
CYP2D6	**1*	252	54.78
	**2*	94	20.43
	**3*	2	0.43
	**4*	51	11.09
	**5*	9	1.96
	**6*	2	0.43
	**10*	7	1.52
	**17*	13	2.83
	**29*	9	1.96
	**35*	10	2.17
	**41*	11	2.39

Table S3. *CYP1A2, CYP3A4, CYP2C9, CYP2C19* and *CYP2D6* genotype frequency.

CYP Gene	*CYP Genotypes*	N	Frequency (%)
CYP1A2	**1/*1*	15	6.38
	**1/*1F*	82	34.89
	**1F/*1F*	138	58.72
CYP3A4	**1/*1*	128	69.95
	**1/*1B*	45	24.59
	**1/*22*	4	2.19
	**1B/*1B*	6	3.28
CYP2C9	**1/*1*	196	84.12
	**1/*2*	24	10.3
	**1/*3*	12	5.15
	**3/*3*	1	0.43
CYP2C19	**1/*1*	153	64.83
	**1/*2*	49	20.76
	**1/*17*	28	11.86
	**2/*17*	1	0.42
	**2/*2*	1	0.42
	**17/*17*	4	1.69
CYP2D6	**1/*1*	74	32.17
	**1/*10*	2	0.87
	**1/*17*	8	3.48
	**1/*2*	45	19.57
	**1/*29*	2	0.87
	**1/*2x2*	1	0.43
	**1/*3*	1	0.43
	**1/*35*	5	2.17
	**1/*4*	18	7.83
	**1/*41*	6	2.61
	**1/*4x2*	3	1.3
	**1/*5*	2	0.87
	**1/*6*	1	0.43
	**17/*29*	1	0.43

**17/*35*	1	0.43
**1x2/*1*	1	0.43
**1x2/*17*	1	0.43
**1x2/*2*	1	0.43
**1x2/*29*	1	0.43
**1x2/*4*	2	0.87
**1x3/*1*	1	0.43
**1x3/*29*	1	0.43
**2/*10*	4	1.74
**2/*2*	11	4.78
**2/*29*	1	0.43
**2/*35*	1	0.43
**2/*4*	11	4.78
**2/*41*	3	1.3
**2/*4x2*	1	0.43
**2x2/*2*	1	0.43
**2x2/*35*	1	0.43
**2x2/*5*	1	0.43
**3/*5*	1	0.43
**35/*41*	1	0.43
**4/*10*	1	0.43
**4/*17*	2	0.87
**4/*29*	2	0.87
**4/*35*	1	0.43
**4/*4*	3	1.3
**4/*41*	1	0.43
**4/*5*	2	0.87
**4/*6*	1	0.43
**5/*29*	1	0.43
**5/*5*	1	0.43

Table S4. Genotype-predicter metabolizers groups frequency.

CYP Gene	CYP Genotype predicted metabolizer groups	N	Frequency (%)
CYP3A4	PM	6	3.28
	IM	49	26.78
	NM	128	69.95
CYP2C9	PM	1	0.43
	IM	36	15.65
	NM	193	83.91
CYP2C19	PM	1	0.42
	IM	50	21.19
	NM	153	64.83
	RM	28	11.86
	UM	4	1.69
CYP2D6	PM	8	3.48
	IM	46	20
	NM	167	72.61
	UM	9	3.91
